# Translation from Microgravity Research to Earth Application

**DOI:** 10.3390/ijms231910995

**Published:** 2022-09-20

**Authors:** Daniela Grimm, Ruth Hemmersbach

**Affiliations:** 1 Department of Biomedicine, Aarhus University, Ole Worms Allé 4, 8000 Aarhus, Denmark; 2 Department of Microgravity and Translational Regenerative Medicine, University Clinic for Plastic, Aesthetic and Hand Surgery, Otto von Guericke University, Pfälzer Str. 2, 39106 Magdeburg, Germany; 3 Research Group “Magdeburger Arbeitsgemeinschaft für Forschung unter Raumfahrt- und Schwerelosigkeitsbedingungen” (MARS), Otto von Guericke University, 39106 Magdeburg, Germany; 4 Department of Gravitational Biology, Institute of Aerospace Medicine, German Aerospace Center, 51147 Cologne, Germany

The topic “Translation from Microgravity Research to Earth Application” comprises publications focusing on space life sciences, gravitational biology and space medicine [1,2,3,4,5,6,7,8,9,10,11,12,13,14]. It covers publications reporting the impact of altered environmental conditions, such as microgravity (µ*g*), cosmic radiation and isolation on organisms down to the level of cells [3,4,5,7,8,10,11,12,13,14]. In addition, the topic collects studies validating causal diagrams of human health risks for spaceflight [1], hypergravity studies [2] and investigations about the impact of extreme isolation in the Antarctica on the human body [6].

µ*g* provides a unique research environment and an opportunity to identify the mechanism of gravity-sensing and related signaling pathways, regulation and adaptation responses at the cellular, tissue and organism level, covering animals, plants and humans [15,16]. µ*g*-research is supported and validated by ground-based studies in µ*g*-analogues and simulations, as well as under increased gravitational (hypergravity) conditions, providing comprehensive and new knowledge on the regulation of cellular and subcellular functioning [17,18,19,20].

Lack of sedimentation as a characteristic of the µ*g*-environment facilitates the assembly of 3D cell constructions and bioprinting with innovative potential applications in tissue and bioengineering techniques [17,21]. A prerequisite for long-lasting deep space missions is the knowledge of the effect of µ*g* on key biological systems, such as the immune, musculoskeletal, cardiovascular, neurosensory, neuroendocrine, excretory and respiratory systems, their functionality and homeostasis, focusing on molecular/cellular processes but also the development of life support systems [22].

There are many indications and novel findings that pathophysiological changes observed during and after spaceflight represent models of chronic diseases known from Earth. An example is postmenopausal osteoporosis. 

This topic provides examples from µ*g*-studies with potential applications to Earth-related issues. It presents seven research articles and seven reviews. 

These 14 excellent papers were published as detailed in Table 1.

This topic covered review articles focusing on the health of astronauts exposed to long-term µ*g* in space. A longer space mission enormously influences the human body and is accompanied with various health concerns such as cardiovascular problems (hypotension, arrhythmias and cardiac atrophy), muscle atrophy, bone loss together with kidney stones owing to calcium release from bone resorption, dysfunction of the immune system, ocular and neurological problems or disturbance of the biological clock, among others [23]. 

Humans in space experience µ*g*, which enhances bone loss by 0.5–1.5% and reduces bone mineral density per month in space. One reason for this finding is, among others, the inhibition of the Wnt/β-catenin signaling pathway [3]. Different types of exercise and pharmacological treatments are available and discussed in [3]. A second review highlighted the different mechanisms and factors regulating the humoral crosstalk between muscle and bone [8]. The authors focused on the interplay between myokines and osteokines and their mutual regulation [8].

Reynolds et al. validated causal diagrams of human health risks for spaceflight and used as an example bone data from rodents [1]. They applied DAGs to determine the risk of bone fractures of rodents in space [1]. Ganse et al. [4] focused on comparable cartilage alterations in humans, animals and cells during spaceflight. The exposure to µ*g* combined with radiation is likely to lead to joint cartilage thinning and degeneration and consequently to osteoarthritis after long-term space missions. Therefore, research on countermeasures is necessary with regard to future deep space exploration adventures to avoid or inhibit the development of osteoarthritis [4]. 

A further contribution studied the effects of µ*g* provided by parabolic flight maneuvers on human chondrocytes [9]. The authors introduced a new experimental setup based on the fluorescent Ca^2+^ reporter CaMPARI2, onboard LED arrays and subsequent microscopic analysis on the ground. CaMPARI2 showed a strong Ca^2+^ response triggered by histamine, but it was not affected by the alternating gravitational load of a parabolic flight [9]. The tested system is suitable for environments with varying accelerations and is useful for future large-scale pathway analyses with pharmacological libraries [9].

One problem of a long-term spaceflight is the isolation of the space travelers, which can be simulated by long-term stays in Antarctica. Feuerecker et al. [6] reported on a one-year expedition in Antarctica. The individual expedition members showed increased or even new allergic reactions to environmental allergens after their return. Long-term confinement in the Antarctic seems to alter immune function, which is in some individuals pronounced after return to the familiar allergen environment [6], an interesting finding which has to be studied in detail in the future. So far, the dysfunction of the immune system of astronauts had been addressed by various studies [23]. Another publication of this topic reported changes in macrophages exposed to the Rotating Wall Vessel for three days [14]. In M0, M1 and M2 phenotypes, s-µ*g* results in a decrease in TNF-expression and an increase in IL-12 and VEGF expression. IL-10 was also significantly increased in M1 and M2, but not M0 macrophages [14]. These data can improve our knowledge about macrophage function in s-µ*g*, but validation under real spaceflight conditions is necessary. Another interesting study showed that collagen type XV is related to endoplasmic reticulum stress and inflammation of adipose tissue [13]. The FAK/integrin β1 signaling pathway and M1 macrophages are involved in this process and should be addressed in mice or rats exposed to s-µ*g* or in space. Sarkar et al. [5] reviewed the current knowledge about bone marrow remodeling and dysfunction of the innate immune system in vitro and in vivo in space and with µ*g*-simulation techniques. The importance of multicellular spheroids to answer immunological questions during and after future spaceflights was extensively discussed [5].

Two research articles focused on neurological changes induced by altered gravity conditions [2,12]. An in vitro study evaluated the impact of hypergravity to potentially modify key features of astrocyte reactivity [2]. Fundamental mechanisms on shape and mobility of astrocytes are affected due to increased gravitational stimulation (hypergravity). Lichterfeld et al. identified an attenuation of key features of astrocyte reactivity due to hypergravity exposure. This finding suggests hypergravity together with live-cell imaging as a tool for future studies with other cell types, organoids, 3D spheroids or ex vivo cultures [2]. The second neurological study used the hindlimb unloading model (HLU) to expose rats to s-µ*g* for seven days and focused on the distribution of monoamines in functional territories of the rat brain [12]. The analyses reveal remodeling of the 5-HT (serotonin; 5-hydroxytryptamine) system alone or in interaction with catecholaminergic systems, notably DA (dopamine). This profile induced by HLU in Long Evans rats is able to confer a transient vulnerability for the development of neuropsychiatric diseases such as mood disorders [12]. Therefore, rehabilitation programs are applicable for space travelers for their return to Earth.

Long-term stays in space put space travelers at risk of developing serious health problems. Three further reviews of this topic discuss the recent knowledge about skin health [7], cardiovascular health problems [10] and cancer [11] for humans in space. An interesting review reports about stressors in space and skin health [7]. Such spaceflight stressors for the skin are µ*g*, ionizing radiation and psychological stress and are associated with skin health problems [7]. To find countermeasures to protect astronaut’s skin, simulation models and their combination have to be developed to study the effects of cosmic radiation, µ*g* and psychological stress hormones. Baran et al. [10] summarized current research and knowledge in the field of space life sciences with a focus on the cardiovascular system in the real and simulated µ*g*-environment. In early µ*g*, the cephalad fluid shift increases the stroke volume (35–46%) and cardiac output (18–41%) in astronauts. Later decreases in arterial pressure are occurring and result in the development of cardiac atrophy in space [10]. Moreover, arrhythmias were reported. In vivo and in vitro models reveal cellular and molecular changes, including alterations in cell shape and endothelial dysfunction [24]. Finally, this topic covers a review about cancer in space [11]. There is still an unclear risk for cancer in astronauts. In vitro studies demonstrated that µ*g* induces multicellular spheroid formation, cytoskeleton rearrangement, gene and protein expression changes and apoptosis [11,25]. Novel OMICs results suggest new biomarkers and drug targets useful to develop effective cancer treatments [25,26,27].

In summary, the excellent papers included in this topic report novel findings in the field of space life sciences research.

We would like to thank the authors who supported this topic. We are convinced that research on the International Space Station, in outer space, in extreme environments as well as methods for simulation of µ*g* in combination with novel molecular biological technologies such as OMICs contribute toward the health protection and treatment of diseases of future space travelers who conquer the universe during deep space exploration missions to Moon and Mars. The results will be also translated to health issues on Earth.

## Figures and Tables

**Table 1:** Contributions to the Topic “Translation from Microgravity Research to Earth Application”.

Author	Title	Topics and Results	Type	Ref.
Reynolds R.J.	Validating causal diagrams of human health risks for space flight: An example using bone data from rodents	NASA Human Systems Risk Board uses causal diagrams (DAGs) DAGs for modeling complex risk systems Risk of bone fracture after exposure to spaceflight in rodents Causal pathways between skeletal unloading and bone strength	Research article	[1]
Lichterfeld Y. et al.	Hypergravity attenuates reactivity in primary murine astrocytes	Primary murine astrocytes exposed to 2 *g* and 10 *g* hypergravity, 0 h, 2.5 h, 5 h, 24–72 h Live-cell imaging: Reduction in spreading rates, migration velocities, and stellation Cytoskeletal changes No apoptosis and no changes in proliferation	Research article	[2]
Baran R. et al.	Microgravity-related changes in bone density and treatment options: A systematic review	µ*g* promotes an increased bone turnover with bone loss Countermeasures: Exercise on treadmills or resistive apparatus, pharmacological treatments with bisphosphonates, RANKL antibody (receptor activator of nuclear factor κβ ligand antibody), proteasome inhibitor, pan-caspase inhibitor, and interleukin-6 monoclonal antibody	Review	[3]
Ganse B. et al.	Joint cartilage in long-duration spaceflight	Simulated (s-) µ*g* (s-µ*g*) (unloading) and radiation exposure: joint degeneration (cartilage thinning, changes in cartilage composition) Limited evidence from space missions—serum biomarker data in only a few astronauts Research in this area is needed as well as suitable countermeasures	Review	[4]
Sarkar R. et al.	In vitro models of bone marrow remodelling and immune dysfunction in space: Present state and future directions	Review about the impact of spaceflight conditions on innate immunity in in vitro and animal models Latest in vitro models of the bone marrow stem cell niche	Review	[5]
Feuerecker M. et al.	One year in the extreme isolation of Antarctica—Is this enough to modulate an “allergic” sensitization?	1-year stay in Antarctica Reports of increased or new allergic reactions to environmental allergens Chip-based multiplex assay: One-third of 39 participants: elevated IgEs against pollen Antarctic long-term confinement can induce an altered immune function, which is pronounced in some participants after return to the familiar allergen environment	Research Article	[6]
Radstake W.W. et al.	Spaceflight stressors and skin health	Skin spaceflight stressors: µ*g*, ionizing radiation and psychological stress Overview of in vitro and in vivo simulation models simulating these stressors	Review	[7]
Lau P. et al.	Dissociation of bone resorption and formation in spaceflight and simulated microgravity: Potential role of myokines and osteokines?	Review of mechanisms and factors regulating the humoral crosstalk between muscle and bone Focus on the interplay between known myokines and osteokines and their mutual regulation	Review	[8]
Hammer A. et al.	Retrograde analysis of calcium signaling by CaMPARI2 shows cytosolic calcium in chondrocytes is unaffected by parabolic flights	75th ESA parabolic flight (PF) campaign: human chondrocytes Fluorescent Ca^2+^ reporter CaMPARI2, onboard LED arrays, and microscopic analysis on ground CaMPARI2 showed a strong Ca^2+^ response triggered by histamine but was not affected by the alternating gravitational load of a (PF)	Research Article	[9]
Baran R. et al.	The cardiovascular system in space: Focus on in vivo and in vitr*o* studies	Early µ*g*: Cephalad fluid shift increases the stroke volume (35–46%) and cardiac output (18–41%). Absence of orthostatic pressure, decrease in arterial pressures and cardiac atrophy in space Cellular and molecular changes include altered cell shape and endothelial dysfunction Human spaceflight is associated with several cardiovascular risk factors µ*g*-platforms are used to study physiological changes to develop countermeasures	Review	[10]
Cortés-Sánchez J. L. et al.	Cancer studies under space conditions: finding answers abroad	Unclear risk for cancer in astronauts µ*g* involved in carcinogenesis µ*g* induces multicellular spheroid formation, cytoskeleton rearrangement, gene and protein expression changes and apoptosis Deleterious radiation effects on cells seem to be accentuated under µ*g* Novel OMICS findings may help to find effective cancer treatments	Review	[11]
Gros A. et al.	Simulated microgravity subtlety changes monoamine function across the rat brain	S-µ*g* (hindlimb unloading) model for a short period (7 days) in Long Evans male rats Detection of monoamines in thirty brain regions S-µ*g* by mobilizing vestibular/motor systems promotes early restricted changes in NA and DA functions that are associated with a high reorganization of monoaminergic systems, notably 5-HT	Research Article	[12]
Li C. et al.	Collagen XV promotes ER stress-induced inflammation through activating integrin β1/FAK signaling pathway and M1 macrophage polarization in adipose tissue	C57BL/6 J male mice and adipocytes model Col XV aggravates adipose tissue ERS; interaction between Col XV and integrin β1 is necessary for activation of FAK; Col XV triggers adipocyte ERS by disrupting intracellular Ca^2+^ homeostasis through IP3R1; IFNβ secretion from adipose tissue induced by ERS plays a role in M1 macrophage polarization; Col XV promotes ERS induced adipose inflammation through FAK/integrin β1 signaling pathway and M1 macrophage polarization in adipose tissue	Research Article	[13]
Ludtka C. et al.	The effects of simulated microgravity on macrophage phenotype	S-µ*g* (Rotating Wall Vessel, 3 days) on M0, M1, and M2 macrophage phenotypes (PT) All PT: decrease in TNF-α expression and an increase in IL-12 and VEGF expression IL-10 was significantly increased in M1 and M2 Insight in phenotypic macrophage function in µ*g*	Research Article	[14]

## Data Availability

Not applicable.
